# Supplementary material for: Content-rich biological network constructed by mining PubMed abstracts
Source: BMC Bioinformatics. 2004 Oct 8;5:147. doi: 10.1186/1471-2105-5-147 (PMC528731; doi:10.1186/1471-2105-5-147)
Supplement: Additional File 2 — The original results of the above study (non-essential files are deleted to keep the file size under the limit set by BMC bioinformatics). [file 1471-2105-5-147-S2.bz2 › chilibotAdditionalFile2/dip10/585ID9311977E10953/html/RNH_ANG.html]

 


 **RNH** and **ANG** 
  
Found 69 abstracts in PubMed, retrieved 10.  
 

 What does Google say? 
 PDF only 
| .edu only 

---

**Interactive relationship** (e.g. stimulation, inhibition, etc)

**Inhibitory relationship**- RNasin, the placental ribonuclease inhibitor  [ **RNH** ] , is known to have antiangiogenic activity through the inhibition of angiogenin  [ **ANG** ]  and basic fibroblast growth factor.  Ref: 11393280 Cancer Gene Ther, 2001
- The cytosolic ribonucleolytic activity of ribonuclease A  [ **ANG** ]  RNase A and other pancreatic type ribonucleases is limited by the presence of excess ribonuclease inhibitor  [ **RNH** ]  RI.  Ref: 11437592 Protein Expr Purif, 2001
**Neutral relationship**- Genetic screen to dissect protein protein interactions ribonuclease inhibitor  [ **RNH** ]  ribonuclease A  [ **ANG** ]  as a model system.  Ref: 12431438 Methods, 2002

**Non-interactive relationship** (e.g. studied together, co-existance, homology, etc.)

- Ribonuclease A  [ **ANG** ]  RNase A and the ribonuclease inhibitor  [ **RNH** ]  protein RI form one of the tightest known protein protein complexes.  Ref: 12069420 Anal Biochem, 2002
- Fluorescence assay for the binding of ribonuclease A  [ **ANG** ]  to the ribonuclease inhibitor  [ **RNH** ]  protein.  Ref: 12069420 Anal Biochem, 2002
- The efficacy of this approach is demonstrated with the ribonuclease inhibitor  [ **RNH** ]  protein and ribonuclease A  [ **ANG** ] , which form a complex with an equilibrium dissociation constant in the femtomolar range.  Ref: 12431438 Methods, 2002
- The nucleotide sequence of the open reading frame shared significant homology with that of the human ribonuclease angiogenin  [ **ANG** ]  inhibitor  [ **RNH** ]  RI.  Ref: 11167794 Br J Haematol, 2001
